# Thermoregulatory correlates of nausea in rats and musk shrews

**DOI:** 10.18632/oncotarget.1732

**Published:** 2014-02-22

**Authors:** Sukonthar Ngampramuan, Matteo Cerri, Flavia Del Vecchio, Joshua J. Corrigan, Amornrat Kamphee, Alexander S. Dragic, John A. Rudd, Andrej A. Romanovsky, Eugene Nalivaiko

**Affiliations:** ^1^ Research Center for Neuroscience and Institute of Molecular Bioscience, Mahidol University, Bangkok, Thailand;; ^2^ Department of Biomedical and Motor Sciences, University of Bologna, Bologna, Italy;; ^3^ FeverLab, Trauma Research, St. Joseph's Hospital and Medical Center, Phoenix, AZ, USA;; ^4^ School of Biomedical Sciences, Chinese University of Hong Kong, Hong Kong, China;; ^5^ School of Biomedical Sciences and Pharmacy, University of Newcastle, Newcastle, NSW, Australia.

**Keywords:** nausea, chemotherapy, temperature, hypothermia

## Abstract

Nausea is a prominent symptom and major cause of complaint for patients receiving anticancer chemo- or radiation therapy. The arsenal of anti-nausea drugs is limited, and their efficacy is questionable. Currently, the development of new compounds with anti-nausea activity is hampered by the lack of physiological correlates of nausea. Physiological correlates are needed because common laboratory rodents lack the vomiting reflex. Furthermore, nausea does not always lead to vomiting. Here, we report the results of studies conducted in four research centers to investigate whether nausea is associated with any specific thermoregulatory symptoms. Two species were studied: the laboratory rat, which has no vomiting reflex, and the house musk shrew (Suncus murinus), which does have a vomiting reflex. In rats, motion sickness was induced by rotating them in their individual cages in the horizontal plane (0.75 Hz, 40 min) and confirmed by reduced food consumption at the onset of dark (active) phase. In 100% of rats tested at three centers, post-rotational sickness was associated with marked (~1.5°C) hypothermia, which was associated with a short-lasting tail-skin vasodilation (skin temperature increased by ~4°C). Pretreatment with ondansetron, a serotonin 5-HT3 receptor antagonist, which is used to treat nausea in patients in chemo- or radiation therapy, attenuated hypothermia by ~30%. In shrews, motion sickness was induced by a cyclical back-and-forth motion (4 cm, 1 Hz, 15 min) and confirmed by the presence of retching and vomiting. In this model, sickness was also accompanied by marked hypothermia (~2°C). Like in rats, the hypothermic response was preceded by transient tail-skin vasodilation. In conclusion, motion sickness is accompanied by hypothermia that involves both autonomic and thermoeffector mechanisms: tail-skin vasodilation and possibly reduction of the interscapular brown adipose tissue activity. These thermoregulatory symptoms may serve as physiological correlates of nausea.

## INTRODUCTION

Approximately half of cancer patients experience nausea and vomiting during the course of their disease, either due to cancer itself or secondary to chemotherapy [1]. Many commonly used chemotherapeutic agents (eg. cyclophosphamide and cisplatin) possess potent emetogenic properties [2]. Nausea and vomiting produced by these and other chemothrerapeutic drugs are among the most severe and feared collateral effects of chemotherapy [3, 4]. These side effects not only dramatically worsen quality of life, but may affect the patients' willingness to continue their anti-cancer treatment. Of interest, nausea has a stronger negative impact on patients' daily life than vomiting [5].

The introduction of the 5-HT_3_ and neurokinin-1 (NK_1_) antagonists substantially improved the management of chemotherapy-induced vomiting [2, 4]. However, paradoxically, their effect on nausea was comparatively modest [6, 7], and it is now becoming recognized that nausea and vomiting represent totally different entities, both from the point of view of their pathophysiology and pharmacotherapy [8, 9]. The relative potencies of anti-emetic drugs to differentially reduce nausea and emesis were not immediately recognized as nausea was only a secondary end-point in most clinical studies. Likewise, most recent pre-clinical studies of novel anti-emetic substances are based on emetic responses, and it is assumed that drugs which suppress retching/vomiting in animals may be equally good in suppressing nausea in humans. As noted, this is not the case. The situation is even more complicated in ‘nausea’ studies performed in common laboratory animals such as rats and mice, as these animal do not have emetic reflex. Here, a researcher's choice for assessing nausea is limited to just a few biological indices with either poor temporal resolution (e.g. reduced locomotion or food intake) or questionable face validity (e.g. pica - kaolin consumption) and specificity (eg. conditioned food aversion) (reviewed in [8]).

This differential action of anti-emetic drugs on nausea and emesis in the clinic led to the realization that there may be different pathways and control systems for nausea and vomiting [8]. Furthermore, it is now clear that the wealth of existing mechanistic knowledge on the neural mechanism of the vomiting reflex, with its circuitry located mainly in the brainstem [8, 10], is not directly applicable to nausea – a subjective experience that presumably originates in some forebrain areas. Consequently, identification of the key transmitter systems and receptors involved in nausea perception is essential for the improved care of the cancer patients. Providing that major methodological obstacle for advancing in this field is lack of adequate animal models for studying nausea [11], development and validation of new potential physiological indices for such studies is of major importance.

In humans, nausea is commonly associated with facial pallor, sweating and gastric awareness [8]. It is less known, but well documented that in humans nausea is also associated with profound disturbances in thermoregulation, including falls in the core body temperature, modified perception of the ambient temperature and preference for cooler environment [12-15]. It is currently unknown whether pro-emetic stimulation elicits similar disturbances in experimental animals, and we have designed our experiments to elucidate this question. Neither subjective perceptions nor sweating can be assessed in conscious rodents. On the other hand, core body temperature, cutaneous temperature, and the preference for ambient temperature are readily measurable, but none have ever been used in preclinical studies of nausea. Our principal hypothesis was that pro-emetic stimulation causes hypothermia and alters behavioral preference for the ambient temperature in rodents.

## RESULTS

### Provocative motion reduced food consumption in rats.

**Fig 1:**
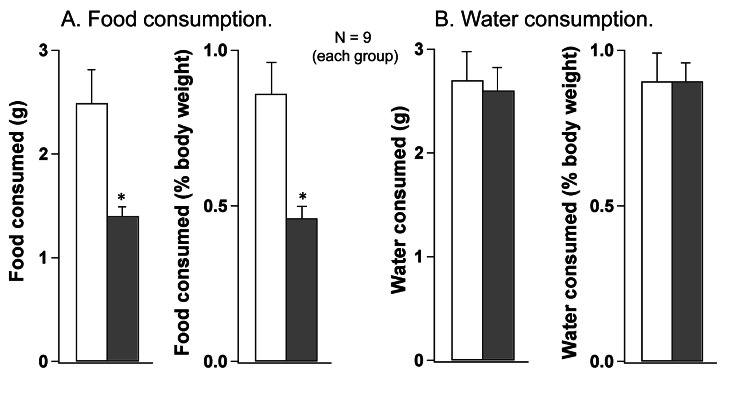
Provocative motion substantially reduced food consumption (A) but had no effect on water consumption (B) in rats Both food and water consumption were determined in absolute values and as a percentage of body weight. White bars - control group; grey bars - after provocative motion. * - significantly different from control (P<0.01).

Provocative motion had substantial and significant anorexic effect (Fig. 1): during 30-min test period, rats subjected to rotation consumed 42% less food compared to controls (1.4±0.1 vs. 2.5±0.3g, respectively; p<0.05); this effect was even larger (49%, or 0.43±0.02 vs. 0.84±0.05 g, respectively; p<0.05) if food consumption was assessed as a percentage of increase in body weight. In contrast, there was no effect of rotation on water consumption (2.6±0.3 vs. 2.7±0.3, or 0.85±0.03 vs. 0.85±0.05%, respectively; p>0.05 for both).

### Provocative motion caused hypothermia in rats.

**Fig 2:**
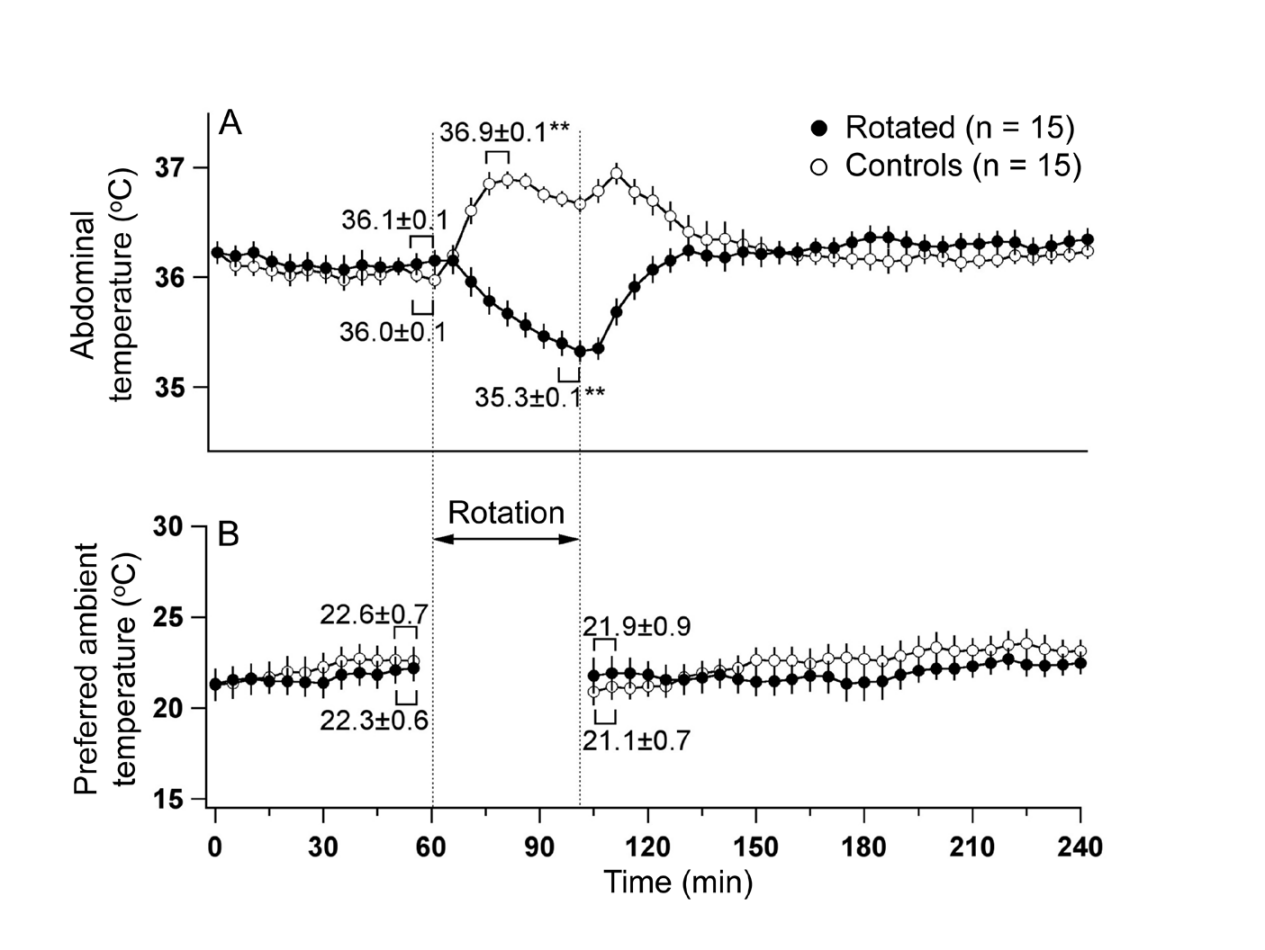
Provocative motion elicited hypothermic responses (A) and had no effect on the preference for the ambient temperature (B) in rats Note that in control animal, moving them to home cage and back to the thermogradient setup provoked rises in body temperature. ** - different from basal values, p<0/01.

During the initial (basal) thermogradient session, there was no difference in basal core body temperature between animals exposed to provocative motion and controls. In all tested rats, rotation caused consistent falls in the core body temperature (Fig. 2A). These falls became apparent within the first 5-7 min of rotation, and did not reach steady state or reverse until rotation was terminated. Following return to the thermogradient, core body temperature returned to the basal level within 30 min. In contrast to hypothermic responses elicited by provocative motion, we observed rises in core body temperature in rats returned to their home cages after the initial thermogradient session. Their temperature started to fall after reaching peak, but slightly increased again following the return back to the thermogradient. Data values for the core body temperature changes and results of statistical tests are presented in Fig. 2A.There were no differences in preferred ambient T between control and experimental conditions (Fig. 2B). Provocative motion had no effect on animals' preferred ambient T for more than 2 h.

### Provocative motion causes an increase in the tail temperature in rats.

**Fig 3:**
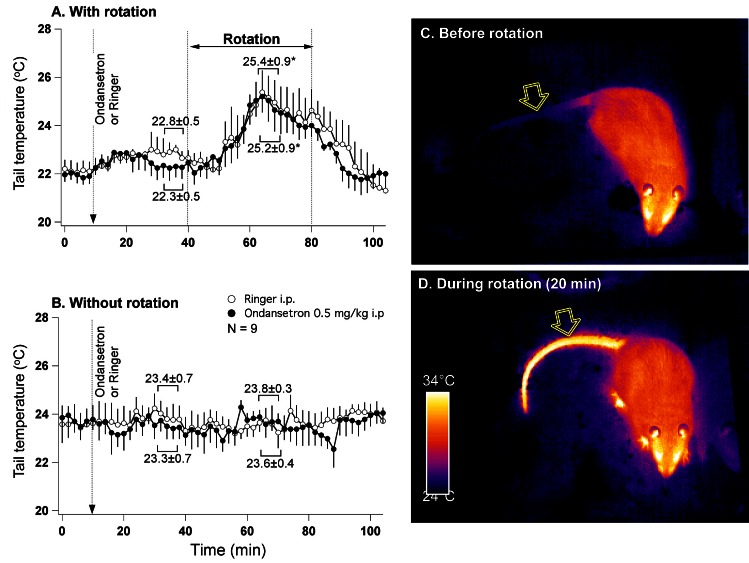
Provocative motion causes rise in tail temperature indicative of vasodilatation A - mean group data (n=7) from the experiment where either ondansetron (0.5 mg/kg) or vehicle were administered i.p. 30 min prior to the onset of rotation. B - mean group data from the control (without rotation) experiment conducted in the same animals. Statistical comparison was made between data obtained during 5 min just prior to the onset of rotation and during maximal tail warming (A) or between corresponding data points (B). * - different from pre-rotation values. Panels C and D represent infrared images (presented in pseudo-colour scale) of a rat before and during rotational stimulation. Note the substantial increase in the temperature of the tail. Arrows indicate tail region used for data collection.

Analysis of infrared images revealed that within 10 minutes from the onset of rotation, tail temperature started to rise, reached peak at about 20 min, and then gradually returned to the baseline (Fig. 3A). Ondansetron had no effect on the amplitude of tail warming (p>0.05). In the control experiment (without rotation), neither Ringer nor ondansetron affected tail temperature (Fig. 3B). Mean data values are also presented in Fig. 3. An example of infrared images obtained in one rat before and 20 min after the onset of rotation are presented in Fig. 3C and D.

### Ondansetron attenuates hypothermic responses to provocative motion in rats.

**Fig 4:**
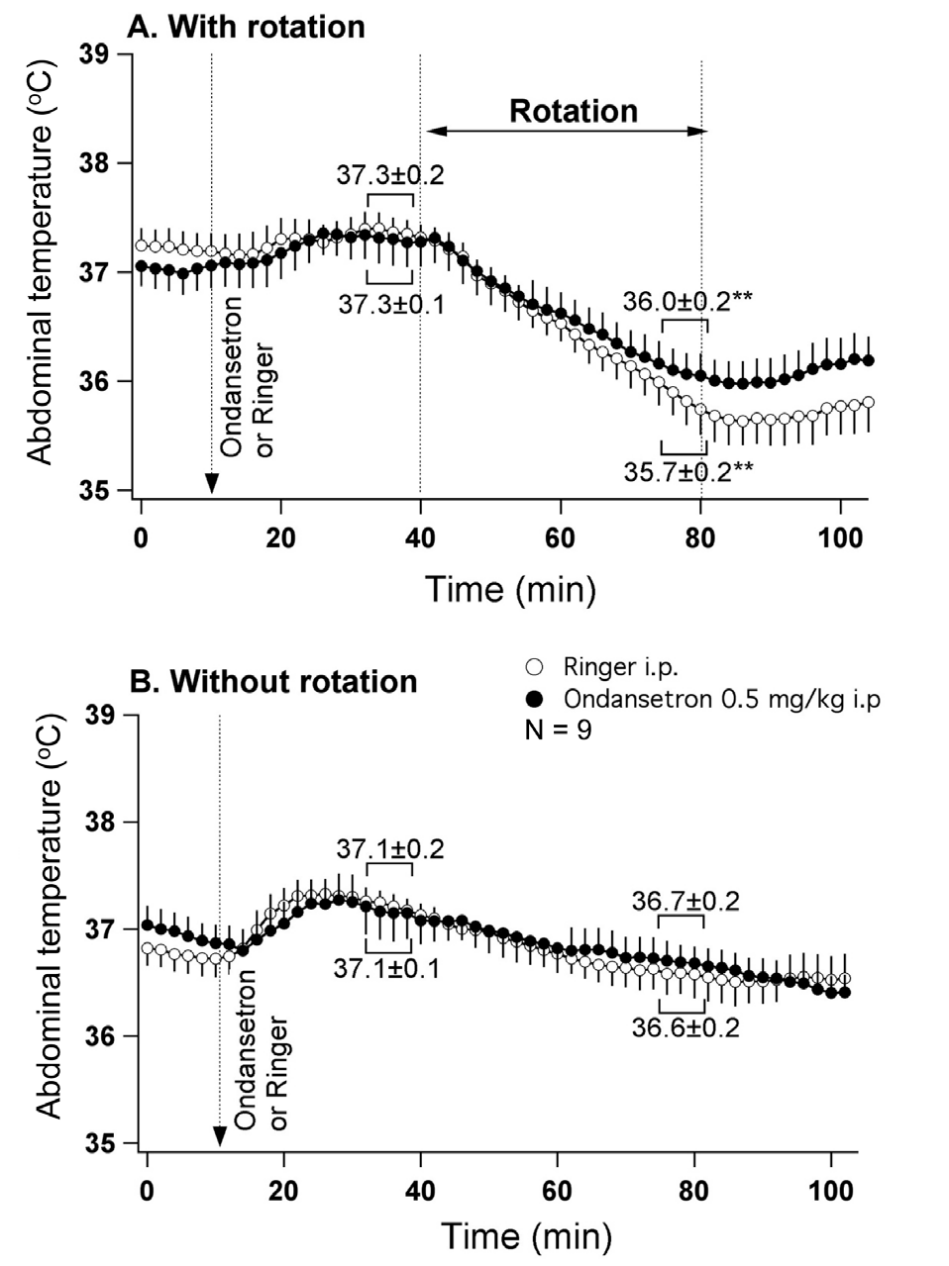
5-HT_3_ receptor blockade slightly reduces hypothermia induced by provocative motion A - mean group data from the experiment where either ondansetron (0.5 mg/kg) or vehicle were administered i.p. 30 min prior to the onset of rotation. B - mean group data from the control (without rotation) experiment conducted in the same animals. Statistical comparison was made between data obtained during 5 min just prior to the onset of rotation and during the last 5 min of rotation (A) or between corresponding data points (B). ** - different from pre-rotation values (p<0.01).

In this group of rats (n=9), provocative motion elicited hypothermic responses that were quantitatively and qualitatively similar to those reported in Exp. 2 above. Pre-treatment with ondansetron partially but significantly reduced this rotation-induced hypothermia (Fig. 4A), with the value of temperature fall post-drug (-1.3±0.2°C) being smaller compared to the post-vehicle value (-1.8±0.2°C; p<0.05). Ondansetron alone did not have any effect on the core body temperature (Fig. 4B).

### Provocative motion affects surface body temperature in musk shrews.

**Fig 5:**
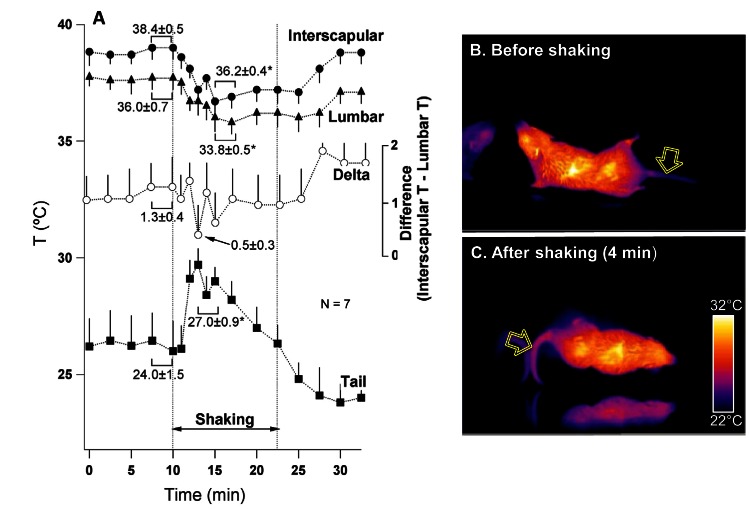
Provocative motion causes rise in tail temperature associate with falls in the interscapular and lumbar temperature in the house shrew (S. *murinus*) A – mean group values for changes in the temperature of interscapular (black circles), lumbar (triangles) and tail (squares) regions and the difference between interscapular and lumbar temperatures (white circles). Statistical comparison was made between data obtained during 2 min just prior to the onset of rotation and 2 min during maximal tail warming. * and ** - different from pre-stimulation, p<0.05 and p<0.01, respectively. Panels B and C represent infrared images (presented in pseudo-colour scale) of a shrew before and during rotational stimulation. Arrows indicate tail region used for data collection.

Our initial intention was to record core body temperature in shrews by means of biotelemetry, similar to the rat study. Unfortunately it appeared that shrews did not tolerate intraperitonial implantation of the transmitters, resulting in high mortality. For this reason we report here only effects of provocative motion on the surface temperature. Similar to previous reports, motion stimuli caused repetitive retching/vomiting episodes in all tested animals (n=7). The mean number of these episodes was 5.7±0.7 (range 4-8 in 15 min), with a mean latency of 195±41 s (range 90-380 s). Analysis of infrared images revealed consistent and substantial reduction in the cutaneous temperature in the interscapular and lumbar areas during provocative motion; this was preceded by a transient increase in the tail temperature that subsequently fell below the basal level. Of note, the difference between the interscapular and the lumbar temperatures tended to fall as the the tail temperature rose. These results are shown in Fig. 5A, with data values presented near the traces. Tail temperature changes were relatively fast, with onset within 1 min of stimulation and with peak at 172±12 s (range 120-240 s). These effects were observed in six of seven animals tested; of note, the non-responder had lower values of basal interscapular, lumbar and tail temperature (36.2, 33.6 and 20.2°C, respectively) compared to five other shrews. An example of infrared images obtained in one shrew before and 6 min after the onset of rotation are presented in Fig. 5B and C.

## DISCUSSION

Our major novel finding is that in rats, provocative motion causes anorexic effect indicative of nausea state associated with a rapid and robust fall in the core body temperature; and that this latter effect is mediated, at least in part, by vasodilatation in the thermoregulatory tail vascular bed. This finding was confirmed in the musk shrew, where tail vasodilation preceded the first vomiting episode, when animals likely experienced nausea. Our observations mirror thermoregulatory changes reported in humans during nausea [12-15]. Together, they form the basis for a new concept – that disturbances in thermoregulation is a core element in the pathophysiology of nausea. Indeed, looking from this angle at the symptoms that accompany nausea in humans, one would immediately realize that they form a “thermoregulatory cluster”. Firstly, and somewhat surprisingly, nausea-related sweating has never been considered as an indication of altered temperature control; meanwhile, it is known that nausea does trigger not only “stress-related” palmar sweating, but also thermoregulatory sweating on the dorsum of the hand [16]. Secondly, it is now documented that in humans experiencing nausea, subjective perception of the ambient temperature as well as subjective preference for it are altered [13]. Lastly, as it was earlier found in humans [12, 14, 15], and as we now report in two different mammalian species, pro-emetic stimuli appear to suppress cold defence, by causing cutaneous vasodilatation leading to heat dissipation and fall in the core body temperature.

### Choice of the pro-emetic intervention.

It is well established that sensitivity to chemotherapy-induced nausea is higher in those patients who are also susceptible to motion sickness and/or with a history of nausea during pregnancy [17, 18]. This suggests that sensitivity to nausea is determined at integrative centers in the brain receiving different inputs. In turn, this allows measuring the sensitivity to one type of emetic stimuli to assess sensitivity to another. This is of major importance for our study as the most direct approach here would be to subject the experimental animals to chemotherapeutic agents only. However, the multiple biological actions of these drugs (e.g. involving gross damage to tissues) and the complexity of relevant pathways (e.g. both peripheral nerve input and input from the area postrema) renders great difficulty in tracing causative links between their initial sites of action and our end-point measurements. For this reason, we induced a ‘nausea-like’ state by provocative motion – a “clean” stimulus, acting through well characterized vestibular pathways, and not involving biochemical effects of chemotherapy. Providing that nausea-related effects of chemotherapy are long-lasting (days), relatively fast recovery from provocative motion is another obvious advantage in addressing mechanisms of nausea.

### Body core and tail temperature as a new physiological correlate of nausea in animals.

Provocative motion has been used previously to examine signs of motion sickness in both musk shrews and rats. In shrews, motion stimuli reliably provoked retching and vomiting [19, 20], similarly to our observation. With the exception of a single article that describes motion-induced alterations in gastric myeloelectric activity [21], all other studies conducted on shrews did not quantify any other indices except emetic episodes. Our work is thus the first to report motion-induced temperature-based effects in this species. Since in humans nausea usually precedes vomiting, we consider the fact that thermoregulatory changes preceded vomiting/retching episodes in shrews as an indirect evidence for a nausea-like state in these animals.

Reduced food consumption following provocative motion has been reported previously in rats [22], and our results are in accord with these earlier data. However, most provocative motion studies conducted in rats were focussed on quantifying pica – an unconventional consumption of kaolin (eg. [23, 24]. While having some predictive validity, this approach has relatively low temporal resolution; morepver, nausea-related pica is absent not only in humans but also in musk shrews and mice [25, 26]. In addition to pica, in rats provocative motion reduces locomotor activity [27] and provokes conditioned taste aversion [28]. There is only one study where the effect of provocative motion on body temperature was examined in rats: Ossenkopp at al. [27] reported that rectal temperature fell by about 2^0^C in animals subjected to horizontal rotation (70 rpm for 30-min). Our work not only reconfirms these earlier finding but also takes it further, by: i) non-invasive measurement of core temperature that eliminate the confounding of the stress response elicited by rectal measurement; ii) providing a real-time temporal course of the temperature fall; and iii) demonstrating that hypothermia is associated with an increase in the tail blood flow.

Similarity between temperature changes elicited by provocative motion in humans, rats and shrews suggests that these mammalian species possibly possess similar neural mechanisms linking nausea to thermoregulation. Consequently, thermoregulatory disturbances may not only be considered as a key accompanying symptom of nausea, but in fact could be used as a valuable physiological correlate of this disorder in experimental animals. This is supported by our finding that falls in body temperature preceded retching/vomiting in shrews: this means that if they experienced sensation akin to nausea prior to these episodes, it was associated with altered temperature control. Of course our proposal requires further validation, in first instance by testing whether observed effects on the temperature are sensitive to drugs used to treat nausea. In the current study, we started this validation from ondansetron, a 5-HT_3_ receptor antagonist with proven antiemetic potency [29]. The drug only moderately attenuated motion-induced hypothermia in our rats. It is unlikely that this poor effect was due to the low dose as we have used a dosage validated in other in vivo bioassays [30]. Rather, if hypothermia reflects a nausea-like state as we suggest, such a poor effect should be expected, providing a recognized dissociation between anti-nausea and antiemetic potency of the drug in humans [29]. Our control experiments (without rotation) confirmed that ondansetron alone does not have any effect on the core body temperature or on the tail temperature. The former observation is in accord with the earlier studies that address this issue [31, 32].

Our negative thermogradient data indicate that contrarily to humans, pro-emetic stimulation does not change rats' preference for the ambient temperature. The reason of this discrepancy is currently unknown, as our thermogradient methodology is highly sensitive and well validated [33]. It may be that in rats the change in the ambient temperature preference does occur, but quickly restores after termination of the stimulus. Presentation of pro-emetic stimuli in the thermogradient may clarify this issue in future experiments.

### Thermoeffectors mediating motion-induced effects on body temperature.

A rise in the tail temperature was associated with falls in body temperature in both rats and shrews. This suggests that in both species, dilatation of the tail vascular bed represented the mechanism underlying heat loss and leading to hypothermia. It appears however that this was not the sole cause of hypothermia observed in our experiments. Lack of ondansetron effect on the motion-induced rise in the tail temperature suggests that the anti-hypothermic action of the drug was not due to preventing tail vasodilation. In rodents, temperature balance is achieved by coordinated regulation of heat loss via cutaneous blood vessels and heat generation by the brown adipose tissue (BAT) (see [34, 35] for reviews). It is thus possible that motion-induced hypothermia was a result of two synergistic effects - increase in the tail blood flow (i.e. increased heat dissipation) and reduction in the BAT thermogenesis, and that ondansetron affected only the latter. The idea of downregulation of the BAT activity by provocative motion is supported, at least in shrews, by observation that not only did the provocative motion reduce interscapular and lumbar skin temperatures, but it also tended to reduce the temperature difference between these two regions, which is indicative of reduced BAT thermogenesis [36].

 Our earlier analysis of thermoeffector threshold data [37] suggests that deep hypothermic responses (such as the ones reported here) occur primarily due to a drastic decrease in the threshold body temperature for BAT thermogenesis (with a much smaller and inconsistent reduction in the threshold temperature for skin vasodilation), thus leading to poikilothermy. The latter, in turn, results in hypothermia – if the ambient temperature is low enough.  The best studied response of this type is LPS-induced hypothermia [36]. Since bacterial endotoxemia is frequently associated with nausea and vomiting (e.g. [38, 39]), it is tempting to suggest that LPS hypothermia and hypothermia induced by pro-emetic stimulation may involve similar mechanisms. It would thus be most interesting to determine the effect of provocative motion on thermoeffector thresholds.

### Neural pathways mediating hypothermia elicited by the provocative motion.

Rotation stimuli used in the present work caused alterations in vestibular and possibly visual sensory inputs, and there must be some area within the brain where this sensory information was converted into the command signals targeting thermoeffectors. Location of central command neurons and efferent pathways controlling thermoeffectors is relatively well understood, at least in rats [34, 35]. Information from central (brain) and peripheral thermosensors is integrated in the preoptic anterior hypothalamus that sends excitatory projection to the dorsomedial hypothalamus, a major integrative centre for autonomic output. Descending presympathetic pathways from the DMH relay in the periaqueductal grey and then in the medullary raphe/parapyramidal area and in the spinal cord, where separate populations of sympathetic neurones control two thermoeffectors – brown adipose tissue responsible for non-shivering thermogenesis and cutaneous vascular bed responsible for heat dissipation/conservation. It is thus clear that there is a limited number of neural targets where neural mechanisms responsible for motion sickness could interfere with the descending thermoregulatory control. Changes in subjective perception of ambient temperature and preference for a cooler environment, commonly known and recently documented effects of motion sickness in humans [13], suggest that this interference occurs quite high in the neuraxis, potentially in the preoptic anrerior hypothalamus.

Knowledge about the pathways controlling tail vascular tone and BAT does not bring any clarity in the question why ondansetron had differential effects on these two thermoeffectors. There is currently no data on involvement of 5-HT3 signalling in either of these pathways. There is however an additional possibility as it is known that provocative motion, similar to other emetic stimuli, reduces locomotion in rats [27]. If ondansetron in our study attenuated this effect, it could be that locomotion-generated heat resulted in reduced hypothermia during motion stimulation in animals treated with the drug. Unfortunately telemetric signal for locomotion cannot be recorded during rotation, and we are unable to confirm or disapprove this possibility.

The most challenging task of our discussion is to provide suggestions about the site of origin of the final neural input that provokes perturbations in the thermoregulatory control. Currently, the dominating theory of motion sickness is the one of sensory conflict; it postulates that when converging vesibular, visual and proprioceptive input patterns differ from the expected sensory pattern stored in memory, spatial orientation is disturbed, and this leads to motion sickness [40]. Until recently, there was no direct data regarding potential anatomical location of neurons generating “sensory mismatch” signal. In an elegant studies conducted in rats [41] demonstrated that this might happen in the hippocampus, whose role in spatial orientation and learning is well recognized. The first human brain imaging study of motion sickness revealed several regions that were activated during the experience of nausea, namely the medial prefrontal cortex, the insular cortex, the amygdala, the putamen and the locus coeruleus [42]; somewhat surprisingly, hippocampus was not among them. Clearly, cited works provide a good framework for future experimental determination of motion sickness-activated inputs to thermoregulatory pathways.

### Physiological significance and perspectives.

Provocative motion could be considered as a stressful intervention, and in this context hypothermia caused by it seems paradoxical providing all other stressors (restraint, social interaction, novelty, footshocks etc.) provoke *hyperthermic* responses in rodents. Ossenkopp [27] presented an interesting explanation of this apparent paradox. Based on the idea that lowing body temperature could represent a defence mechanism against intoxication and on the Treisman's theory that aversive sensation of nausea which accompanies motion sickness is a by-product of evolutionary developed defence against ingested neurotoxins [43], authors postulated that motion sickness-induced hypothermia is a manifestation of this evolutionary beneficial reaction. Its high evolutionary relevance might explain the fact that provocative motion not only led to a fall in the abdominal temperature compared to baseline, but in addition counteracted hyperthermic responses provoked by animal handling (Fig. 2A).

As we stated in the Introduction, the major obstacle in preclinical studies of nausea is the lack of ability to assess its cardinal symptoms (subjective experience and sweating) in experimental animals. The only established biochemical marker of nausea in humans, elevated plasma vasopressin [44, 45], has been confirmed in cats [46] and ferrets [47] but not in rats [44, 48]. Consequently, rodent studies of motion sickness-induced nausea have to rely on indirect indices, often with poor temporal resolution (eg. locomotor activity, food consumption) or limited face validity (eg. pica that, in addition, is species specific [25, 26]). Quantifying retching or vomiting in species possessing emetic reflex (eg. shrews) now appears not to be an ideal solution for nausea studies as here the major difficulty is in the totally different neural substrates responsible for nausea and for vomiting. It is most likely that relying on emetic responses in preclinical studies is the major reason for pharmacological dissociation of current anti-emetics that efficiently suppress vomiting but are less potent in preventing nausea [9, 29].

We believe that our current findings, in combination with the solid evidence obtained in humans and discussed above, represent a firm basis for the claim that altered thermoregulation is a core pathophysiological element of nausea in mammals. Consequently, assessing temperature-related indices in experimental animals subjected to pro-emetic stimuli, including chemotherapeutic agents, may represent a promising novel approach for determining brain neural circuits responsible for nausea, and for assessing its pharmacological sensitivity.

## METHODS

### Animals and experimental protocols.

Investigation has been conducted in accordance with the ethical standards and according to the Declaration of Helsinki and according to national and international guidelines and has been approved by the authors' institutional review board. Experiments 1 and 2 were conducted at St. Joseph's Hospital and Medical Center (Phoenix, AZ, USA); Experiment 3 – at Mahidol University (Bangkok, Thailand) and Experiment 4 - at the University of Bologna (Italy). All rats were adult males of Wistar strain, weighing 220-270 g. Experiment 5 was conducted on the adult male house musk shrews (*Suncus murinus*) at the Chinese University of Hong Kong.

In Experiment 1, we studied the effect of provocative motion on food and water consumption in rats. All food and water was removed from the animals' cages at the beginning of the light cycle (6:00 AM), and animal weights were recorded. Rats were either rotated on a turntable at 0.75 Hz (experimental group, n=9) or not rotated (control group, n=9) for 40 min just prior to the start of the dark cycle (6:00 PM). Animals were then returned to their home cages where pre-weighed water and standard rat chow were made available to them. Food and water were then weighed 30 min after the onset of the dark cycle.

In Experiment 2, we studied the effect of provocative motion on the core body temperature and on preferred ambient temperature (T_amb_) in another group of rats. Each animal's body temperature was recorded using an implantable datalogger (SubCue, Calgary, Alberta, Canada), which was stitched to the inner abdominal wall via midline laparotomy under ketamine-xylazine-acepromazine anesthesia (55.6, 5.5, and 1.1 mg/kg i.p. respectively) and antibiotic protection (enrofloxacin, 5 mg/kg). Animals were allowed to recover for 3 days prior to experimentation. On the day of the experiment, rats were placed inside a thermogradient apparatus (previously described by [33]) to assess baseline preferred T_amb_. The thermogradient apparatus consisted of six 217-cm-long aluminum channels that run between two water tanks. Two electric heating units (PolyScience, Niles, IL, USA) heated the water inside one tank to maintain a T_amb_ of 30^o^C inside the channels at the “warm” end, whereas the water tank at the “cold” end was constantly perfused with 10% ethylene glycol by an external-circulation cooling pump (PolyScience) to maintain a T_amb_ of 15^o^C inside the channels at the opposite end. By manipulating the water temperatures at either end, a near-linear temperature gradient of 0.07^o^C/cm was maintained along each of the six channels. T_amb_ in the thermogradient was continuously monitored via five evenly-spaced (50cm) thermocouples per channel. The thermocouples were fed into a TempScan 1000A receiver (Omega, Stamford, CT, USA), which relayed temperature data to the TempWindows software program (Omega) in real-time. A high-definition video camera (Panasonic WV-CP284, Panasonic, Kadoma, Osaka, Japan), positioned directly overhead, captured each animal's location inside the thermogradient and relayed it to the TopScan software (Cleversys, Reston, VA, USA) for tracking along the established temperature gradient. Rats were then removed, individually housed in their home cages, and placed on a turntable. The turntable was rotated at 0.75 Hz (experimental group, n=15) or not rotated (control group, n=15) for 40 min. Immediately thereafter, rats were returned to the thermogradient apparatus for 3 hours. All animals were well-adapted to the experimental setups prior to experimentation. No drugs were administered during this experiment.

In Experiment 3 we studied the effects of provocative motion on the tail temperature in uninstrumented rats. The protocol (recording timing, drug injections and rotation parameters) was identical to Experiment 3. For assessing tail temperature before and during provocative motion, infrared video images were acquired using a thermoimager (FLIR-A20, Flir Systems, Wilsonville, OR, USA). There were 9 rats in this group.

In experiment 4, we tested whether hypothermic effect of provocative motion is sensitive to antiemetic drug ondansetron. Telemetric transmitters (TA11CA-F40, Data Sciences International, USA) were aseptically implanted into the peritoneal cavity under isoflurane (2% in O_2_) anaesthesia, and animals were left to recover for 1 week; they were housed individually during this time. On the day of experiment, transmitters were turned on, home cages were placed on a turntable that was stationary, and baseline recording of the core body temperature was obtained for 1 h. At the middle of this period (i.e. at 30 min), animals received i.p. injection of either antiemetic ondansetron (0.5 mg/kg in 0.5 ml Ringer) or vehicle (0.5 ml Ringer). Thirty minutes later, the turntable was switched on, and rotation was maintained for 40 min at 0.75 Hz, This protocol was repeated one week later, with a counter-balanced administration of ondansetron or vehicle. During subsequent week 3 and 4, we determined whether there were any effects of the drug or vehicle alone on the temperature. For this purpose, the protocol was repeated again once a week, but without rotation. There were 9 animals in this experiment. Ondansetron was from Sigma (USA).

In Experiment 5, we studied effects of provocative motion on the surface body and tail temperature in adult house musk shrews (*Suncus murinus* – an insectivore possessing the vomiting reflex; N = 7). Animals were housed in groups of 4 per cage. On the day of experiment, each animal was moved to a new clean cage and remained in it for 40 min for habituation prior to recording. A cage with an animal was placed on a laboratory shaker, with an infrared camera (KC500, Keii, China) fixed above the cage. A 15-min basal recording was followed by 15-min of provocative motion (1 Hz, 4 cm linear displacement). We also quantified the number of retching/vomiting episodes and their latencies. We did not administer drugs in this experiment. The ambient temperature for all experiments was 20-21^o^C.

### Data acquisition, analysis and statistical evaluation

The dataloggers (Exp. 2) sampled core body temperature every 3 min; a similar sampling rate was used in assessing the preferable ambient temperature. Radiotelemetric temperature data (Exp. 3) was sampled at 1 Hz and acquired using MacLab-8s data acquisition system (ADInstruments, Sydney, Australia), and then reduced by averaging within every 2-min interval. Infrared images from rats (Exp. 4) were collected from individual frames at 2-min intervals; tail temperature was assessed at the distal region of the proximal third of the tail. Infrared images from shrews (Exp. 5) were also collected from individual frames at 2-min intervals. Using proprietary SmartIRMNet software (Keii, China), we then determined the superficial skin temperature from three areas: interscapular region, bottom of the trunk at the midline, and tail area 1 cm distal from the base. Both cameras have built-in self-calibration capabilities.

For statistical comparison, data were selected as following: Exp. 2, body T – mean of the two last points just before rotation vs. mean of the two points at maximum (for control animals) or at minimum (rotated animals); Exp. 2, thermogradient - mean of the two last points just before rotation vs. mean of the two first points post-rotation; Exp. 3, rotation condition - mean of the four last points just before rotation vs. mean of the two four points during maximal rise in the tail T; Exp. 3, control – corresponding time intervals; Exp. 4, rotation condition - mean of the four last points just before rotation vs. mean of the two four points during maximal fall in the abdominal; Exp. 4, control – corresponding time intervals; Exp. 5 mean of the two last points just before rotation vs. mean of the two four points during maximal rise or fall in each parameter. Statistical significance for the differences in water consumtion was performed usin unpaired Student's t-test. Statistical differences in the core body and the cutaneous temperatures were assessed by means of two-way ANOVA (treatment vs. time) with Bonferroni correction, with p<0.05 being the threshold for significance.­

## References

[1] Warr DG (2008). Chemotherapy-and cancer-related nausea and vomiting. Current oncology.

[2] Di Maio M, Bria E, Banna GL, Puglisi F, Garassino MC, Lorusso D, Perrone F (2013). Prevention of chemotherapy-induced nausea and vomiting and the role of neurokinin 1 inhibitors: from guidelines to clinical practice in solid tumors. Anti-cancer drugs.

[3] Lindley C, McCune JS, Thomason TE, Lauder D, Sauls A, Adkins S, Sawyer WT (1999). Perception of chemotherapy side effects cancer versus noncancer patients. Cancer practice.

[4] de Boer-Dennert M, de Wit R, Schmitz PI, Djontono J, v Beurden V, Stoter G, Verweij J (1997). Patient perceptions of the side-effects of chemotherapy: the influence of 5HT3 antagonists. British journal of cancer.

[5] Bloechl-Daum B, Deuson RR, Mavros P, Hansen M, Herrstedt J (2006). Delayed nausea and vomiting continue to reduce patients' quality of life after highly and moderately emetogenic chemotherapy despite antiemetic treatment. Journal of clinical oncology : official journal of the American Society of Clinical Oncology.

[6] Foubert J, Vaessen G (2005). Nausea: the neglected symptom?. European journal of oncology nursing : the official journal of European Oncology Nursing Society.

[7] Navari RM (2013). Management of chemotherapy-induced nausea and vomiting : focus on newer agents and new uses for older agents. Drugs.

[8] Stern R, Koch K, Andrews P (2011). Nausea.

[9] ASHP (1999). ASHP Therapeutic Guidelines on the Pharmacologic Management of Nausea and Vomiting in Adult and Pediatric Patients Receiving Chemotherapy or Radiation Therapy or Undergoing Surgery. American journal of health-system pharmacy : AJHP : official journal of the American Society of Health-System Pharmacists.

[10] Hornby PJ (2001). Central neurocircuitry associated with emesis. The American journal of medicine.

[11] Sanger GJ, Andrews PL (2006). Treatment of nausea and vomiting: gaps in our knowledge. Autonomic neuroscience : basic & clinical.

[12] Nobel G, Eiken O, Tribukait A, Kolegard R, Mekjavic IB (2006). Motion sickness increases the risk of accidental hypothermia. European journal of applied physiology.

[13] Nobel G, Tribukait A, Mekjavic IB, Eiken O (2012). Effects of motion sickness on thermoregulatory responses in a thermoneutral air environment. European journal of applied physiology.

[14] Mekjavic IB, Tipton MJ, Gennser M, Eiken O (2001). Motion sickness potentiates core cooling during immersion in humans. The Journal of physiology.

[15] Cheung B, Nakashima AM, Hofer KD (2011). Various anti-motion sickness drugs and core body temperature changes. Aviation, space, and environmental medicine.

[16] McClure JA, Fregly AR, Molina E, Graybiel A (1972). Response from arousal and thermal sweat areas during motion sickness. Aerospace medicine.

[17] Morrow GR (1985). The effect of a susceptibility to motion sickness on the side effects of cancer chemotherapy. Cancer.

[18] Roscoe JA, Bushunow P, Morrow GR, Hickok JT, Kuebler PJ, Jacobs A, Banerjee TK (2004). Patient expectation is a strong predictor of severe nausea after chemotherapy: a University of Rochester Community Clinical Oncology Program study of patients with breast carcinoma. Cancer.

[19] Ueno S, Matsuki N, Saito H (1988). Suncus murinus as a new experimental model for motion sickness. Life sciences.

[20] Rudd JA, Ngan MP, Wai MK (1999). Inhibition of emesis by tachykinin NK1 receptor antagonists in Suncus murinus (house musk shrew). European journal of pharmacology.

[21] Percie du Sert N, Chu KM, Wai MK, Rudd JA, Andrews PL (2010). Telemetry in a motion-sickness model implicates the abdominal vagus in motion-induced gastric dysrhythmia. Experimental physiology.

[22] Riccio DC, Thach JS (1968). Response suppression produced by vestibular stimulation in the rat. Journal of the experimental analysis of behavior.

[23] Mitchell D, Laycock JD, Stephens WF (1977). Motion sickness-induced pica in the rat. The American journal of clinical nutrition.

[24] McCaffrey RJ (1985). Appropriateness of kaolin consumption as an index of motion sickness in the rat. Physiology & behavior.

[25] Yamamoto K, Ngan MP, Takeda N, Yamatodani A, Rudd JA (2004). Differential activity of drugs to induce emesis and pica behavior in Suncus murinus (house musk shrew) and rats. Physiology & behavior.

[26] Liu YL, Malik N, Sanger GJ, Friedman MI, Andrews PL (2005). Pica--a model of nausea? Species differences in response to cisplatin. Physiology & behavior.

[27] Ossenkopp KP, Rabi YJ, Eckel LA, Hargreaves EL (1994). Reductions in body temperature and spontaneous activity in rats exposed to horizontal rotation: abolition following chemical labyrinthectomy. Physiology & behavior.

[28] Ossenkopp KP (1983). Area postrema lesions in rats enhance the magnitude of body rotation-induced conditioned taste aversions. Behavioral and neural biology.

[29] Olver IN (2005). Update on anti-emetics for chemotherapy-induced emesis. Internal medicine journal.

[30] Tuerke KJ, Winters BD, Parker LA (2012). Ondansetron interferes with unconditioned lying-on belly and acquisition of conditioned gaping induced by LiCl as models of nausea-induced behaviors in rats. Physiology & behavior.

[31] Kandasamy SB (1997). Effect of ondansetron and ICS 205-930 on radiation-induced hypothermia in rats. Radiation research.

[32] Mazzola-Pomietto P, Aulakh CS, Murphy DL (1995). Temperature, food intake, and locomotor activity effects of a 5-HT3 receptor agonist and two 5-HT3 receptor antagonists in rats. Psychopharmacology.

[33] Almeida MC, Steiner AA, Branco LG, Romanovsky AA (2006). Neural substrate of cold-seeking behavior in endotoxin shock. PloS one.

[34] Romanovsky AA (2007). Thermoregulation: some concepts have changed. Functional architecture of the thermoregulatory system. American journal of physiology Regulatory, integrative and comparative physiology.

[35] Morrison SF, Nakamura K (2011). Central neural pathways for thermoregulation. Frontiers in bioscience.

[36] Marks A, Vianna DM, Carrive P (2009). Nonshivering thermogenesis without interscapular brown adipose tissue involvement during conditioned fear in the rat. American journal of physiology Regulatory, integrative and comparative physiology.

[37] Romanovsky AA (2004). Do fever and anapyrexia exist? Analysis of set point-based definitions. American journal of physiology Regulatory, integrative and comparative physiology.

[38] Todd JK (1985). Staphylococcal toxin syndromes. Annual review of medicine.

[39] Brock-Utne JG, Gaffin SL (1989). Endotoxins and anti-endotoxins (their relevance to the anaesthetist and the intensive care specialist). Anaesthesia and intensive care.

[40] Reason J, Brand J (1975). Motion sickness.

[41] Aitake M, Hori E, Matsumoto J, Umeno K, Fukuda M, Ono T, Nishijo H (2011). Sensory mismatch induces autonomic responses associated with hippocampal theta waves in rats. Behavioural brain research.

[42] Napadow V, Sheehan JD, Kim J, Lacount LT, Park K, Kaptchuk TJ, Rosen BR, Kuo B (2013). The brain circuitry underlying the temporal evolution of nausea in humans. Cerebral cortex.

[43] Treisman M (1977). Motion sickness: an evolutionary hypothesis. Science.

[44] Rowe JW, Shelton RL, Helderman JH, Vestal RE, Robertson GL (1979). Influence of the emetic reflex on vasopressin release in man. Kidney international.

[45] Fisher RD, Rentschler RE, Nelson JC, Godfrey TE, Wilbur DW (1982). Elevation of plasma antidiuretic hormones (ADH) associated with chemotherapy-induced emesis in man. Cancer treatment reports.

[46] Fox RA, Keil LC, Daunton NG, Crampton GH, Lucot J (1987). Vasopressin and motion sickness in cats. Aviation, space, and environmental medicine.

[47] Hawthorn J, Andrews PL, Ang VT, Jenkins JS (1988). Differential release of vasopressin and oxytocin in response to abdominal vagal afferent stimulation or apomorphine in the ferret. Brain research.

[48] Verbalis JG, McHale CM, Gardiner TW, Stricker EM (1986). Oxytocin and vasopressin secretion in response to stimuli producing learned taste aversions in rats. Behavioral neuroscience.

